# Successful haploidentical hematopoietic stem cell transplantation for activated phosphoinositide 3-kinase δ syndrome: Case report and literature review

**DOI:** 10.1097/MD.0000000000032816

**Published:** 2023-02-03

**Authors:** Xiaolan Yang, Rui Xi, Jiaofeng Bai, Yaozhu Pan

**Affiliations:** a Department of Hematology, The 940th Hospital of Joint Logistics Support Force of Chinese People’s Liberation Army, Lanzhou, China.

**Keywords:** activated PI3Kd syndrome, HSCT, PIK3CD, primary immunodeficiency (PID)

## Abstract

Activated phosphoinositide 3-kinase δ syndrome (APDS), a recently described primary immunodeficiency,is caused by autosomal dominant mutation in the phosphatidylinositol-4,5-bisphosphate 3-kinase catalytic subunit delta(PIK3CD) gene encoding the p110δ catalytic subunit of PI3Kδ (APDS1) or the PIK3R1 gene that encodes the p85α regulatory subunit of PI3Kδ (APDS2). Gain-of-function mutation of PIK3CD in APDS1 leads to p110δ hyperactivity, with the result of the hyperphosphorylation of downstream mediators of Akt and mammalian target of rapamycin that cause a series of clinical symptoms. Few cases with APDS were reported in Asia.

**Patient concerns:**

We report a 6-year-old patient with a recurrent respiratory infection, cryptosporidium enteritis, lymphoproliferation, high serum immunoglobulin-M level, anemia, and inverted CD4+/CD8+ ratio. The whole exome sequencing confirmed a heterozygous missense mutation c.3061G>A（p.E1021K）in patient and her mother. Her mutant gene is inherited from her mother, but her mother has not any clinical symptoms.

**Diagnoses:**

Activated phosphoinositide 3-kinase δ syndrome.

**Interventions:**

The patient was received immunoglobulin (Ig) replacement therapy, antibiotics, and rapamycin treatment. Through effectively controlling infection and optimal timing of transplantation by adjusting the conditioning regimen, haploidentical Hematopoietic Stem Cell Transplantation(haplo-HSCT) from her brother was successfully performed.

**Outcomes:**

The patient is in good condiion with a good quality of life after 20 months of follow-up.

**Lessons:**

We reported a rare APDS1 case with PIK3CD^E1021K^ gene mutation, Successfully treated with haplo-HSCT. This case provided a reference for treating APDS with haplo-HSCT.

## 1. Introduction

Activated phosphoinositide 3-kinase δ syndrome (APDS) is a recently described primary immunodeficiency (PID) caused by autosomal dominant mutation in the phosphatidylinositol-4, 5-bisphosphate 3-kinase catalytic subunit delta (PIK3CD) gene encoding the p110δ catalytic subunit of PI3Kδ (APDS1)^[1,2]^ or the PIK3R1 gene that encodes the p85α regulatory subunit of PI3Kδ (APDS2).^[3,4]^ Gain-of-function mutation of PIK3CD in APDS1 lead to p110δ hyperactivity, with the hyperphosphorylation of downstream mediators of Akt and mammalian target of rapamycin (mTOR).^[1]^ APDS is a PID characterized by recurrent respiratory infections, lymphoproliferation, increased lymphoma susceptibility, hyper immunoglobin (Ig)-M syndrome, poor antibody production, and chronic Epstein Barr virus (EBV) and cytomegalovirus (CMV) infections.^[5]^ Few APDS cases were reported in Asia.

Here, we reported a classical APDS case with PIK3CD^E1021K^ gene mutation with recurrent respiratory tract infections, *Cryptosporidium enteritis*, high serum level of IgM, anemia, lymphoproliferation, and inverted CD4^+^/CD8^+^ ratio, successfully treated with haploidentical hematopoietic stem cell transplantation (haplo-HSCT) from her brother.

## 2. Case presentation

A 6-year-old girl was admitted to our hospital because of persistent anemia and diarrhea for 3 years. At the age of 3, she was noticed to be pale, weak, and had chronic diarrhea, and her hemoglobin fell to 60 to 70 g/L, the abdominal B ultrasound revealed mild hepatomegaly and severe splenomegaly. During these years, she had recurrent episodes of rhinosinusitis, pharyngotonsillitis, and pneumonia. She had ever been treated with glucocorticoid and blood transfusion, but there was no improvement. She was referred to our hospital on July 15, 2018. Physical examination showed moderate anemia, bilateral cervical lymphadenopathy, mild hepatomegaly, and severe splenomegaly. Her parents were healthy. The patient was a second-pregnancy child with no family history of immune-deficiency nor recurrent infections or serious diseases.

Laboratory examinations showed: white blood cells 20.74 × 10^9^/L, hemoglobin 68g/L, platelets 211 × 10^9^/L; serum lactate dehydrogenase was 369 IU/L with normal liver and kidney function. She had a high serum level of IgM (2270 mg/dL), a low level of IgA (54.8 mg/dL), and IgG (315 mg/dL). Coombs test was negative. The peripheral blood lymphocyte subsets consisted of reduced CD4^+^T cells and CD19^+^ B cells, and the CD4^+^/CD8^+^ index was 0.59. The human immunodeficiency virus, EBV, CMV, and toxoplasmosis were negative. Cervical lymph node biopsy suggested reactive proliferation. Cryptosporidium enteritis was diagnosed by microbiological examination and colonoscopy. Bone marrow biopsy showed erythrocytosis. Lung computed tomography scan showed increased lung texture.

**Figure 1.**
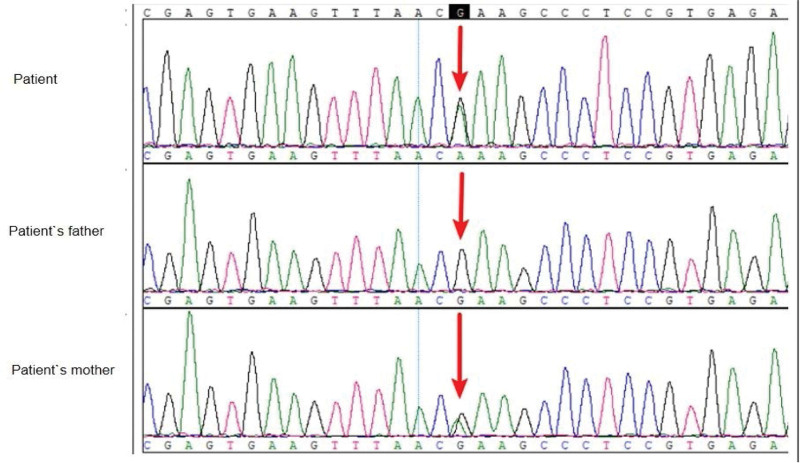
PIK3CD gene sequences of the patient and her parents (the patient and her mother: PIK3CD:c.3061G > A:p.E1021K). PIK3CD = phosphatidylinositol-4, 5-bisphosphate 3-kinase catalytic subunit delta.

The whole exome sequencing of venous blood was performed on the patient and her parents. A heterozygous missense mutation c.3061G > A (p.E1021K) of PIK3CD gene was revealed in this patient and her mother (see Fig. 1). Her mother has not had any clinical symptoms with normal serum level of IgM and normal CD4^+^/CD8^+^ ratio.

The patient was diagnosed with activated PI3Kδ syndrome with PIK3CD^E1021K^ gene mutation. She received Ig replacement therapy (IRT) (400 mg/kg/mo), antimicrobial therapy (nitazoxanide and azithromycin for 2 weeks), and rapamycin treatment (1 mg once daily). During the following 6 months, the patient was still infected intermittently, the level of IgM was still high, a decision to have an HSCT was made and the human leukocyte antigen matching between her brother and her is haploidentical, and the PI3KCD gene of his brother was normal.

On February 27, 2021, haplo-HSCT from her brother was undergone, the conditioning regimens were fludarabine 30mg/m^2^ per day for 3 consecutive days (from day −8 to day −6), busulfan 3.2 mg/kg per day (from day −8 to day −6) and cyclophosphamide 40 mg/kg per day (from days −5 to −2); ATG 7.5 mg/kg per day (from days −4 to −2); On day 0, the patient received 5.8 × 10^6^ CD34^+^ cells/kg and 8.1 × 10^8^ mononuclear cells/kg. To prevent graft versus host disease, patients received intravenous cyclosporine A from day −1 before transplantation (to reach the target level of 200 ng/mL or more), intravenous methotrexate 15 mg/kg m² on day 1, and subsequently 10 mg/m² on days 3, 6, and 11. Granulocyte engraftment occurred on day +12, and platelets engrafted on day +14. Bone marrow biopsy showed normalization of trilineage hematopoiesis on day +30, with chimerism of 98.7%.

The patient was regularly checked in the outpatient clinic and developed grades 2 acute graft versus host disease at +3 months post-transplantation, which improved after immunosuppressive treatment. Up to date, she has been followed up for 20 months, she is well and has not subsequently experienced a deep-seated infection, chronic diarrhea, or fungal infection. The original abdominal cavity enlarged lymph node returned to normal, the level of IgG gradually rises, the level of IgM gradually decreased post HSCT, and it returned to normal at +5 month (Table 1). Inverted CD4/CD8 ratio returns to normal post-HSCT (Table 2).

**Table 1:** The change of immunoglobulin before and after transplantation.

Time	IgG	IgA	IgM
	(751–1560 mg/dL)	(82–453 mg/dL)	(46–304 mg/dL)
2019/2	33	62.5	2270
2019/8	236	50	2140
2021/2	369	50.1	2060
2021/3 (0 mo*)	318	32.4	1731
2021/5 (+2 mo*)	638	10.7	25.3
2021/8 (+5 mo*)	869	43.2	122
2022/2 (+11 mo*)	1060	31.9	182
2022/8 (+17 mo*)	951	43.4	239

Ig = immunoglobulin.

* The month post hematopoietic stem cell transplantation (HSCT).

**Table 2:** The change of CD4^+^/CD8^+^ ratio before and after transplantation.

Time	CD4^+^T cell (%)	CD8^+^T cell (%)	CD4^+^/CD8^+^ ratio
2019/2	27.2	51.2	0.5
2019/8	28.9	41	0.7
2021/2	21.3	44.9	0.5
2021/3 (0 mo*)	28.9	23.6	1.2
2021/5 (+2 mo*)	34.4	23.6	1.5
2021/8 (+5 mo*)	43.6	31	1.4
2022/2 (+11 mo*)	43.2	36.8	1.4

* The month post hematopoietic stem cell transplantation (HSCT).

Informed written consent was obtained from the patient’s parents for the publication of this case report and accompanying images.

Ethics board approval and consent were obtained for this work from the Ethics Committee at the 940th Hospital of Joint Logistic Support Force of Chinese People’s Liberation Army, China (940hec-2019036).

## 3. Discussion

PIDs are a widely heterogeneous group of disorders that lead to immune dysfunction and increase susceptibility to infections. To date, >400 underlying genetic deficits have been shown to lead to various PIDs.^[6]^ APDS is a recently reported PIDS. Since the first APDS with germline heterozygous gain-of-function mutations in PIK3CD gene was identified, >10 different activating missense mutations of PIK3CD gene have been revealed in APDS1.^[7]^ The PIK3CD gene mutation is by far the most frequent APDS mutation with a frequency of 85%.^[8]^ The PIK3CD gene mutation could increase the activation of PI3K signaling by enhancing the association of the catalytic subunits with membranes and facilitating the phosphorylation of PIP2.^[9]^

Variants that cause APDS are unlikely to be found in a healthy group because APDS is a rare monogenic disease with high penetrance.

For example, out of the 53 cases from 30 APDS1 families, only 1 carrier of the E1021K mutation was healthy.^[10]^ The E1021K mutation in our patient was inherited from her mother, but the mother had no clinical symptoms. The manifestations of APDS, even with the same mutation, are highly variable (just as in our case), which range from asymptomatic patients to those suffering from a severe immunodeficiency leading to early death, and others with lymphoproliferation and malignancy.^[11]^

APDS patients may develop immunodeficient characteristics including a high rate of recurrent respiratory infections caused by bacterial pathogens, severe, recurrent herpes virus infections such as CMV, EBV infections, and immuno-deregulatory disorders including autoimmunity, lymphoma, non-neoplastic lymphoproliferation, and neurodevelopment delay.^[10,11]^ The treatment for APDS should be carefully tailored to meet each individual patient`s needs for its great clinical heterogeneity. Some asymptomatic carriers only require simple observation, while patients with recurrent bacterial or viral infections may need supportive therapy such as prophylactic antibiotics and IRT, or HSCT.^[11]^ IRT is reported to be useful for APDS patients to reduce respiratory infections.^[10,12]^ Recently Maccari et al^[13]^ reported the initial data of the ESID APDS registry, they found the significant benefit of mTOR inhibitor rapamycin for APDS and associated non-neoplastic lymphoproliferative disease. Eight out of 25 patients had complete response and 11/25 had partial response after the therapy of rapamycin. However, rapamycin was found less beneficial to treat APDS-related cytopenia and gastrointestinal disease. The long-term benefits and risks of rapamycin remain unclear. With greater efficacy and fewer adverse effects, selective PI3Kδ inhibitors (leniolisib) have the potential to provide a targeted treatment option for patients with APDS. Rao et al^[14]^ reported a clinical trial that involved 6 APDS patients with lymphadenopathy and splenomegaly, all of them received 12-week leniolisib, a significant reduction in lymphadenopathy and spleen size was seen in all patients, and no patient experienced significant side effects. Leniolisib may be a preferred optimal for APDS-related lymphoproliferation.

HSCT can be curative for clinical symptoms of pre-HSCT. Nademi et al^[15]^ published a series of patients receiving HSCT for APDS with a survival rate of 81.8% (9/11), 5 received matched (10/10) unrelated donor stem cells, 4 had a matched sibling donor, and 2 had mismatched unrelated donors, and 8 of 9 surviving patients had complete remission of symptoms and discontinued intravenous Ig. Dimitrova D and Nademi Z^[16]^ et al retrospectively collected 57 patients with APDS1/2 who underwent HSCT, with a median follow-up of 2.3 years, 2-year OS and graft failure-free survival probabilities were 86% and 68%, respectively, and there was no significant difference by APDS1 versus APDS2, donor type, or conditioning intensity. The 2-year incidence of graft failure following the first HCT was 17% overall but 42% if mTORi were used in the first year post-HCT, compared with 9% without mTORi. Similarly, the 2-year cumulative incidence of unplanned donor cell infusion was overall 28%. Post-HCT mTORi use may confer an advantage to residual host cells, promoting graft instability. Graft failure, graft instability, and poor graft function requiring unplanned donor cell infusion were major barriers to successful HCT.

In our case, the child had a history of respiratory tract infection, recurrent diarrhea, and high IgM immunophenotype, and genetic testing revealed a heterozygous causative mutation carrying the PIK3CD gene, which confirmed the diagnosis of APDS. After haplo-HSCT, she is well with phenotype reversal and has a resolution of recurrent respiratory infections and Ig replacement requirement during the 20-month follow-up.

In conclusion, APDS is a rare case with PIK3CD gene mutation. Haplo-HSCT can be recommended as an alternative treatment option for APDS patients without a matched donor.

## Author contributions

**Conceptualization:** Jiaofeng Bai, Rui Xi.

**Data curation:** Jiaofeng Bai, Rui Xi.

**Formal analysis:** Rui Xi.

**Supervision:** Jiaofeng Bai.

**Writing-original draft:** Xiaolan Yang, Rui Xi.

**Writing-original draft:** Yaozhu Pan.
